# Intrinsic and extrinsic mechanisms regulating satellite cell function

**DOI:** 10.1242/dev.114223

**Published:** 2015-05-01

**Authors:** Nicolas A. Dumont, Yu Xin Wang, Michael A. Rudnicki

**Affiliations:** 1 Sprott Centre for Stem Cell Research, Ottawa Hospital Research Institute, Ottawa, Ontario, CanadaK1H 8L6; 2 Faculty of Medicine, Department of Cellular and Molecular Medicine, University of Ottawa, Ottawa, Ontario, CanadaK1H 8M5

**Keywords:** Satellite cell, Muscle stem cell, Myogenesis, Quiescence, Cell cycle regulation, Asymmetric division, Self-renewal, Skeletal muscle, Regeneration, Aging

## Abstract

Muscle stem cells, termed satellite cells, are crucial for skeletal muscle growth and regeneration. In healthy adult muscle, satellite cells are quiescent but poised for activation. During muscle regeneration, activated satellite cells transiently re-enter the cell cycle to proliferate and subsequently exit the cell cycle to differentiate or self-renew. Recent studies have demonstrated that satellite cells are heterogeneous and that subpopulations of satellite stem cells are able to perform asymmetric divisions to generate myogenic progenitors or symmetric divisions to expand the satellite cell pool. Thus, a complex balance between extrinsic cues and intrinsic regulatory mechanisms is needed to tightly control satellite cell cycle progression and cell fate determination. Defects in satellite cell regulation or in their niche, as observed in degenerative conditions such as aging, can impair muscle regeneration. Here, we review recent discoveries of the intrinsic and extrinsic factors that regulate satellite cell behaviour in regenerating and degenerating muscles.

## Abstract (precis)

**Summary:** This Review discusses how satellite stem cell behaviour is regulated during regeneration and degeneration by a complex balance between extrinsic cues and intrinsic regulatory mechanisms.

## Introduction

**Fig. 1.**
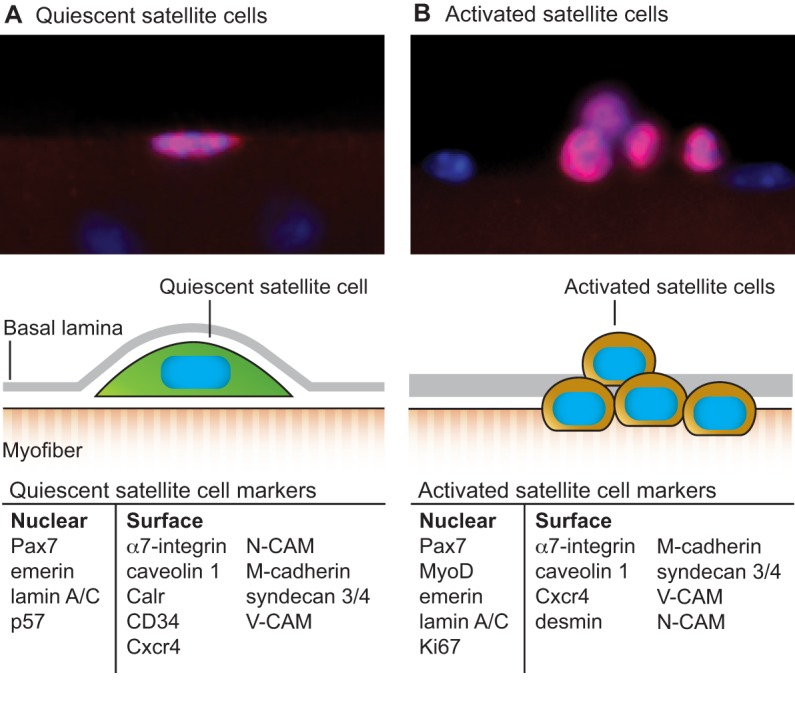
**The satellite cell and its niche.** Satellite cells are located juxtaposed to mature muscle fibers in a quiescent state. Upon activation by extrinsic factors, satellite cells re-enter the cell cycle and proliferate to generate sufficient numbers of progeny to form new myofibers. Micrographs show Pax7-expressing (red) quiescent (A) and activated (B) satellite cells on cultured single myofibers. The schematics beneath represent the quiescent and activated satellite cells in their niche and enumerate the nuclear and surface molecular markers associated with each state.

**Fig. 2.**
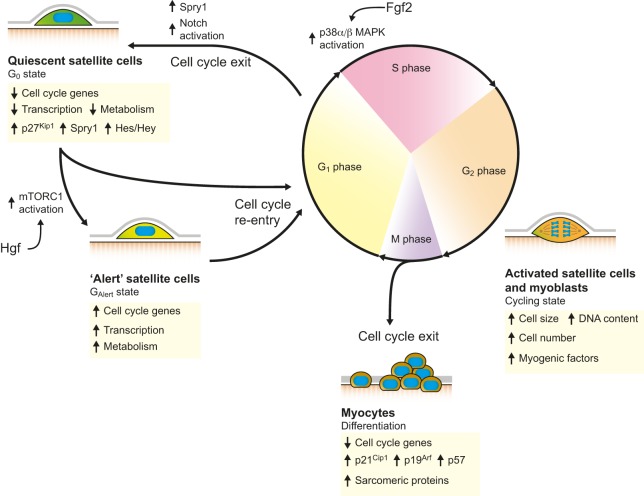
**Regulation of the cell cycle in satellite cells.** In resting conditions, intrinsic regulators of the cell cycle maintain satellite cells in a reversible and quiescent G_0_ state. Activated satellite cells then re-enter the cell cycle, either directly or via an intermediate state referred to as G_Alert_. After activation, satellite cells can exit the cell cycle and return to quiescence by upregulating Spry1 or by increasing Notch signalling. Proliferating myoblasts also exit the cell cycle to differentiate into myocytes and progress into the myogenic lineage.

Skeletal muscles are made up of numerous multinucleated myofibers that possess the contractile machinery to generate movement. Skeletal muscles also contain a population of small mononucleated muscle stem cells, termed satellite cells, that represent 2-10% of total myonuclei (2×10^5^ to 1×10^6^ cells/g muscle) (Hawke and Garry, 2001; White et al., 2010). Satellite cells are located in a specialized niche between the myofiber sarcolemma and the surrounding extracellular matrix (ECM), which is termed the basal lamina (Fig. 1). In resting adult muscles, satellite cells are quiescent and are characterized by the expression of the paired box protein Pax7. Following injury or growth stimulus, satellite cells become activated and express the myogenic regulatory factors Myf5 and/or MyoD (also known as Myod1). Activated satellite cells, which are called myoblasts, proliferate massively to generate the myogenic progenitors needed for muscle regeneration (Bischoff, 1986a; Hurme and Kalimo, 1992; Zammit et al., 2002). Thereafter, myoblasts downregulate the expression of Pax7 and upregulate the expression of factors such as myogenin (Myog) and myogenic regulatory factor 4 (MRF4; also known as Myf6) to exit the cell cycle, differentiate and fuse to form newly regenerating myofibers (Fig. 2). Satellite cells are responsible for the tremendous regenerative capacity of skeletal muscles; many studies have demonstrated a complete lack of regeneration in adult skeletal muscles depleted of Pax7-expressing satellite cells (Günther et al., 2013; Lepper et al., 2011; Sambasivan et al., 2011; von Maltzahn et al., 2013).

Following muscle injury, activated satellite cells give rise to the myogenic progenitors needed to form new myofibers, but they also need to self-renew to maintain the satellite cell pool. Repeated injury experiments have shown that satellite cell numbers remain constant even after multiple traumas (Shi and Garry, 2006). The ability of satellite cells to appropriately balance quiescence, self-renewal and commitment is thus crucial to ensure the life-long maintenance of skeletal muscle. Importantly, many studies now support the notion that satellite cells are heterogeneous and comprise a subpopulation of committed satellite cells that are more prone to progress into the myogenic lineage and a subpopulation of satellite stem cells that are predisposed to undergo self-renewal (Beauchamp et al., 2000; Collins et al., 2005; Kuang et al., 2007; Rocheteau et al., 2012; Sacco et al., 2008).

In recent years, many intrinsic and extrinsic factors governing satellite cell functions have been discovered. Together, these studies demonstrate that the fine-tuned regulation of cell cycle repressors and activators is crucial to coordinate the different states of satellite cells. In this Review, we analyze the intrinsic mechanisms that control satellite cell cycle progression throughout the different stages of adult myogenesis. We also describe how intrinsic mechanisms and extrinsic signals from the satellite cell niche cooperate to influence satellite cell fate decisions, in particular self-renewal versus myogenic commitment. Finally, we discuss the intrinsic defects of aged satellite cells and describe how these deficits modulate the ability of satellite cells to appropriately regulate the cell cycle and cell fate decisions, which ultimately affects their regenerative capacity.

## The development and heterogeneity of satellite cells

During development, the myogenic progenitors that generate skeletal muscles arise from the dorsal portion of the somites, known as the dermomyotome (Bentzinger et al., 2012). Embryonic myogenic progenitors are characterized by expression of the paired box proteins Pax3 and Pax7 (Bober et al., 1994; Relaix et al., 2004; Seale et al., 2000). Embryonic myogenesis in these muscle precursor cells follows the hierarchical activation of Myf5 and/or MyoD, followed by Myog and MRF4, resulting in the formation of embryonic muscle compartments (Hasty et al., 1993; Rawls et al., 1998; Rudnicki et al., 1993; Zhang et al., 1995). In parallel to myofiber formation, a subpopulation of myogenic precursor cells that do not express the MRFs and maintain Pax3/Pax7 expression is observed adjacent to the myofibers late during mouse fetal development, at around E16.5-18.5 (Kassar-Duchossoy et al., 2005). It is hypothesized that these cells give rise to the satellite cell population found in adult muscle. Although adult satellite cells do not express MyoD in resting conditions, the use of a MyoD-iCre mouse strain with a lineage-tracing reporter allele suggests that the progenitors of essentially all adult satellite cells transcribed *MyoD* prenatally (Kanisicak et al., 2009). Contrary to MyoD expression, distinct populations of Myf5-positive and Myf5-negative satellite cells are present in adult muscles, as observed in Myf5-nlacZ reporter mice and by the direct detection of Myf5 protein levels (Beauchamp et al., 2000; Gayraud-Morel et al., 2012; Kuang et al., 2007). To determine whether the Myf5-negative satellite cells represent a distinct population that has never expressed Myf5 during development, Myf5-Cre/ROSA26-YFP mice, in which cells expressing Myf5 and their progeny are permanently labelled with yellow fluorescent protein (YFP), were used. These analyses revealed that a subpopulation of ∼10% of total satellite cells never expresses Myf5 during development (Kuang et al., 2007).

This heterogeneity in the developmental origins of satellite cells raises the possibility that subsets of satellite cells have self-renewal capacity and act as muscle stem cells. Accordingly, in Myf5-Cre/ROSA26-YFP mice, the YFP-negative satellite cells possess higher self-renewal ability than YFP-positive cells, which are more prone to commit into myogenic progenitors. Transplantation experiments clearly highlight the differences between satellite stem cell (YFP^−^) and committed satellite cell (YFP^+^) subpopulations, with the former resulting in long-term engraftment into the transplanted muscle while the latter leading to differentiation and fusion to the host myofibers (Kuang et al., 2007). Using Pax7-nGFP mice, it was shown that, under regenerating conditions, activated satellite cells expressing higher levels of Pax7 are less prone to commitment than those expressing lower levels of Pax7 (Rocheteau et al., 2012). Experiments on TetO-H2B-GFP mice, which are used to report proliferative history, showed that some satellite cells retain the expression of H2B-GFP (termed label-retaining cells, or LRCs), whereas others lose the labelling over time (non-LRCs) (Chakkalakal et al., 2014). LRCs represent a population of satellite cells that are able to self-renew, whereas non-LRCs are committed to differentiation. The findings regarding LRCs in the satellite cell pool agrees with previous experiments that defined satellite cell heterogeneity by cell cycle kinetics and with other recent studies that suggest better self-renewal capacity in slow-dividing cells (Ono et al., 2012; Schultz, 1996).

Together, these studies demonstrate that satellite cells are in fact a heterogeneous population that can be divided into subpopulations of committed satellite cells (i.e. cells that are predisposed to progress through the myogenic lineage once activated) as well as a subpopulation of satellite stem cells (i.e. cells that are able to self-renew and maintain the satellite cell pool). However, whether the satellite stem cell populations identified with the various reporter mouse models represent the same or different subsets of satellite stem cells remains to be determined.

## Cell cycle regulation in satellite cells

Muscle regeneration is characterized by different myogenic stages, namely: activation, proliferation, differentiation, and self-renewal/return to quiescence. Careful regulation of the cell cycle is essential to ensure appropriate progression through these various overlapping states. The following sections describe the intrinsic mechanisms and extrinsic signals that regulate the satellite cell cycle.

### Satellite cell quiescence

In resting adult muscles, satellite cells exist in a dormant state known as quiescence or the reversible G_0_ state (Fig. 2). The ability of satellite cells to maintain quiescence in the resting state is essential for the long-term conservation of the satellite cell pool (Bjornson et al., 2012; Mourikis et al., 2012). This quiescent state is distinct from the cell cycle exit observed prior to differentiation, the most notable difference being its reversibility, which allows cells to return to a proliferative state in response to injury. The rapid cell cycle re-entry of satellite cells after injury suggests that the quiescent state is highly regulated and represents a ‘ready’ state that is primed for activation. Microarray analyses revealed that more than 500 genes are highly upregulated in quiescent satellite cells compared with cycling myoblasts (Fukada et al., 2007; Liu et al., 2013). Within this quiescence signature are negative regulators of the cell cycle, including cyclin-dependent kinase inhibitors 1B (*Cdkn1b*; also known as *p27* or *p27^Kip1^*) and 1C (*Cdkn1c*; also known as *p57* or *p57^Kip2^*), the retinoblastoma tumor suppressor protein (*Rb*; also known as *Rb1*), regulator of G-protein signalling 2 and 5 (*Rgs2*, *Rgs5*), peripheral myelin protein 22 (*Pmp22*), and the negative regulator of fibroblast growth factor (FGF) signalling sprouty 1 (*Spry1*). These quiescence genes act in concert to prevent the precocious activation of quiescent satellite cells, and it was shown that the conditional knockout of p27^Kip1^ or Rb protein results in aberrant satellite cell activation and proliferation (Chakkalakal et al., 2014; Hosoyama et al., 2011). Importantly, impairments in the ability of satellite cells to maintain quiescence reduce self-renewal capacity and muscle regeneration.

Given the highly regulated state of satellite cell quiescence, it is important to consider the transcriptional regulation of quiescence genes. In recent years, the Notch pathway has emerged as a master regulator of satellite cell quiescence. Notch signalling activity is higher in quiescent satellite cells than in activated myogenic cells (Mourikis et al., 2012). This increased activity could be mediated by the interaction between the Notch ligand Delta1 (delta-like 1 in mouse), which is expressed by myofibers, and the Notch receptor (and its co-receptor syndecan 3), which is present on satellite cells (Conboy and Rando, 2002; Pisconti et al., 2010). In addition, expression of the Notch1 and Notch3 receptors is activated by the forkhead transcription factor Foxo3, which is also enriched in quiescent satellite cells (Gopinath et al., 2014). Upon binding of Notch ligand to its receptor, the Notch intracellular domain (NICD) is released and translocates into the nucleus where it interacts with recombining binding protein suppressor of hairless (Rbpj) and triggers the transcription of various genes, including those in the Hes and Hey families. Accordingly, the conditional depletion of Rbpj in satellite cells or the double knockout of *Hey1* and *HeyL* results in spontaneous activation of quiescent satellite cells, impairment in self-renewal and depletion of the satellite cell pool (Bjornson et al., 2012; Fukada et al., 2011; Mourikis et al., 2012). Interestingly, genetic models of Notch inactivation also lead to the spontaneous differentiation of satellite cells, suggesting that Notch has a dual role in maintaining the myogenic progenitor state. During regeneration, downregulation of Notch is mandatory to allow myogenic cell lineage progression (Brack et al., 2008).

Satellite cell quiescence is also maintained by microRNAs (miRNAs). The overall requirement of miRNAs in satellite cell quiescence is exemplified by their precocious activation after deletion of Dicer, the enzyme involved in pre-miRNA hairpin cleavage (Cheung et al., 2012). Although our overall understanding of miRNA regulation in satellite cells is limited, the functions of specific families of miRNAs were recently discovered. Muscle-specific miRNAs, such as miR-1/206 and miR-133, are thought to maintain the myogenic program and facilitate the transition into differentiation (Ma et al., 2015; Townley-Tilson et al., 2010). Although miR-1 and miR-206 share identical seed sequences, the independently regulated expression of the three genomic copy variants (miR-1-1/miR-133a-2, miR-1-2/miR-133a-1, miR-206/miR-133b clusters) confounds the exact genetic requirement for any one particular locus (Besser et al., 2014; Boettger et al., 2014; Wystub et al., 2013). However, the lack of skeletal muscle phenotypes in the knockout of any single miR-1/206/133 cluster suggests that they share overlapping functions or that alternative mechanisms could circumvent their function in these specific knockout models. Other miRNAs regulate the cell cycle and prevent premature differentiation. For instance, miR-489 is highly enriched in quiescent satellite cells where it post-transcriptionally represses the oncogene *Dek* and prevents cell cycle entry (Cheung et al., 2012). Additionally, the ability of satellite cells to maintain quiescence but be poised for activation was shown to be at least partially due to the sequestration of miRNAs together with mRNAs. Indeed, the storage of miR-31 together with *Myf5* in satellite cell mRNP granules prevents the translation of this myogenic regulatory factor and repress myogenic commitment (Crist et al., 2012).

### Activation and cell cycle re-entry

Mitogenic factors liberated following injury drive quiescent satellite cells to re-enter the cell cycle (Bischoff, 1986b, 1990). Recent analyses of systemic effects of muscle injury revealed an intermediate state between the G_0_ quiescent and activated cell states, termed G_Alert_ (Fig. 2). It was shown that, following muscle injury by intramuscular BaCl_2_ injection, satellite cells from the contralateral uninjured leg are phenotypically different from classical quiescent cells (Rodgers et al., 2014). Interestingly, satellite cells from contralateral legs are larger, have more intracellular ATP and higher metabolic activity than quiescent satellite cells. Intriguingly, these ‘alert’ satellite cells express a number of cell cycle genes similar to those expressed in activated satellite cells, but they do not take up BrdU (a proliferation marker) or enter the cell cycle. This intermediate G_Alert_ state allows satellite cells to perform their first division faster than satellite cells in the G_0_ state (Rodgers et al., 2014). This pre-activation state is particularly important because the first cell cycle takes much longer to complete than subsequent cell cycles, which indicates that exit from quiescence is a relatively slow process (Bischoff, 1986b; Siegel et al., 2011). Ultimately, satellite cells in the G_Alert_ state have greater regenerative potential. Although the contralateral leg is subject to overloading in these types of injury experiment, it was suggested that the release of hepatocyte growth factor (Hgf) at the site of injury could have a systemic effect and activate mTOR signalling in distant quiescent satellite cells, thereby driving their entry into the G_Alert_ state (Rodgers et al., 2014). This hypothesis is consistent with a previous study showing that Hgf is present in crushed muscle extract and that direct injection of Hgf into uninjured muscle leads to satellite cell activation (Tatsumi et al., 1998). This implies the existence of a system-wide response mechanism that primes satellite cells to become activated in a regenerative environment. Hence, it will be interesting to assess whether this response is associated with exercise-induced training effects or is possibly altered in pathological conditions.

It is well characterized that the vast majority of satellite cells and myogenic progenitors proliferate in a coordinated effort in response to injury. A peak in myoblast proliferation is reached between the second and fifth days after cardiotoxin-induced muscle injury (Murphy et al., 2014; Ogawa et al., 2014; Rodgers et al., 2014). Damaged muscles release various growth factors that activate signalling pathways involved in satellite cell cycle entry (Anderson et al., 1995). For instance, insulin-like growth factor (Igf1) was shown to inactivate the transcription factor Foxo1, leading to downregulation of the cell cycle inhibitor p27^Kip1^ and ultimately resulting in cell cycle entry (Chakravarthy et al., 2000; Machida et al., 2003). Fgf2 is also highly expressed in regenerating muscle and is known to activate different MAPK pathways in satellite cells (Yablonka-Reuveni et al., 1999). For example, studies of isolated myofibers have shown that p38α/β MAPK (Mapk14/11) is activated and quickly translocates into the nucleus of activated satellite cells after myofiber isolation and, accordingly, the inhibition of p38α/β MAPK impairs the ability of satellite cells to enter the cell cycle (Jones et al., 2005). In addition, activation of the ERK1/2 (Mapk3/1) pathway by Fgf2 was shown to be crucial for the G1 to S phase transition in satellite cells (Jones et al., 2001). JNK, another MAPK signalling pathway, was also shown to trigger activation of cyclin D1 and promote cell cycle progression (Perdiguero et al., 2007). Furthermore, microarray analyses confirmed that a number of cell cycle regulators, including cyclins A, B, D, E, F and G, are enriched in cultured myoblasts compared with freshly sorted quiescent satellite cells (Fukada et al., 2007).

### Cell cycle exit and the return to quiescence

Once activated, satellite cells are then instructed to undergo differentiation (into myocytes) or to return to quiescence, two processes that involve cell cycle exit (Fig. 2). This cell cycle exit requires the upregulation of specific cyclin-dependent kinase inhibitors. Whereas the return to quiescence requires p27^Kip1^, differentiation requires the concerted upregulation of p21^Cip1^ (Cdkn1a), p19^Arf^ (Cdkn2a) and p57 (Cao et al., 2003; Chakkalakal et al., 2014; Pajcini et al., 2010).

Recent evidence has partially unravelled the mechanisms that allow satellite cells to return to quiescence. The tyrosine kinase signalling inhibitor Spry1 is expressed at low levels in activated satellite cells, but is upregulated in satellite cells returning to quiescence (Shea et al., 2010). Spry1 promotes cell cycle exit by inhibiting the ERK signalling pathway. Accordingly, satellite cells from *Spry1* null mice show an impaired ability to return to quiescence, resulting in a decreased satellite cell pool after muscle injury (Shea et al., 2010). Activation of Notch signalling is also crucial for satellite cells to return to quiescence. During the asymmetric division of activated satellite stem cells, the daughter cell that is committed to differentiate expresses high levels of Delta1, whereas the daughter satellite stem cell expresses the Notch3 receptor, which results in the activation of Notch signalling in this cell and promotes its return to quiescence (Kuang et al., 2007). Notch activation also inhibits the expression of MyoD and promotes the expression of Pax7, thus encouraging maintenance of the primitive satellite cell state (Bröhl et al., 2012; Wen et al., 2012). Moreover, the loss of Notch leads to a failure in the homing of satellite cells to their sublaminar position due to the inability of these cells to produce a variety of ECM proteins, such as collagen isoforms, integrin alpha 7, and cell adhesion molecules including components of the Dystroglycan complex (Bröhl et al., 2012). This suggests that, in order for satellite cells to return to quiescence *in vivo*, cell-cell adhesion and ECM deposition are necessary checkpoints that are ultimately intrinsically regulated through autocrine mechanisms.

## The regulation of satellite cell fate decisions

**Fig. 3.**
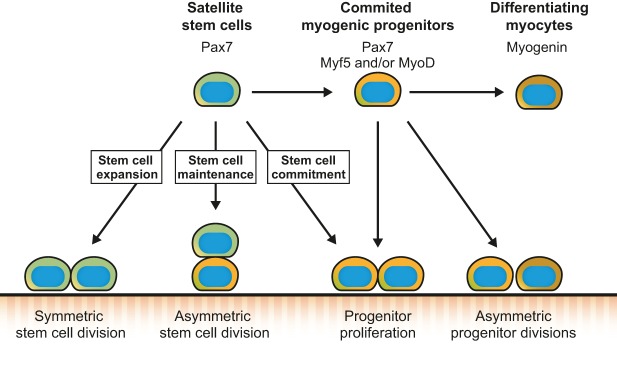
**Satellite cell fate decisions.** Activated satellite stem cells (Pax7^+^, Myf5^–^, MyoD^–^) can undergo symmetric divisions to expand the satellite stem cell population, or asymmetric divisions to maintain the stem cell population and generate myogenic progenitors. Satellite cells can also commit to the myogenic lineage and proliferate to give rise to committed myogenic progenitors (Pax7^+^, Myf5^+^ and/or MyoD^+^). Myogenic progenitors are able to asymmetrically divide or directly differentiate into myocytes (Myog^+^), which fuse and form new myofibers.

In addition to re-entering the cell cycle, activated satellite cells must also determine the cell fate of their daughter cells, in particular whether they self-renew or generate myogenic progenitors. The analysis of MRF expression in proliferating satellite cells revealed asymmetric expression of determination factors, including Myf5, MyoD and Myog, in the daughter cells of subsets of satellite cell divisions (Kuang et al., 2007; Shinin et al., 2006; Troy et al., 2012). It is believed that stem cell subpopulations are able to undergo both symmetric and asymmetric modes of self-renewal to maintain satellite cell numbers through repetitive rounds of regeneration. Tracking the activation of Myf5 with Myf5-Cre/ROSA26-YFP mice revealed that YFP-negative satellite stem cells are able to perform symmetric divisions, which give rise to two identical daughter cells that will self-renew the satellite stem cell pool (Kuang et al., 2007). Alternatively, satellite stem cells can perform asymmetric divisions, which generate one stem cell and one committed daughter cell that will progress through the myogenic lineage (Fig. 3) (Kuang et al., 2007). This section will describe how cell fate decisions are determined during stem cell divisions, and how the niche and intrinsic processes, such as polarity and metabolism, regulate this process.

### Symmetric and asymmetric divisions

In Myf5-Cre/ROSA26-YFP mice ∼30-40% of the first divisions of YFP-negative cells are symmetric, while the remaining divisions are asymmetric (Le Grand et al., 2009). Similar percentages were observed in Pax7-nGFP^Hi^ satellite cells (Yennek et al., 2014). Symmetric division is promoted by activation of the planar cell polarity (PCP) pathway, which leads to the symmetric distribution of polarity effectors such as Vangl2 in daughter cells (Le Grand et al., 2009). Knockdown of Vangl2 in isolated myofibers results in increased numbers of Myog-positive progenitors but decreases total satellite cell numbers, indicating a role for symmetric division in the self-renewal and expansion of the satellite cell pool (Le Grand et al., 2009).

Asymmetric division is characterized by the segregation of different cell fate determinants into the daughter cells. During the asymmetric division of Myf5-negative satellite stem cells, the Notch3 receptor is enriched in the daughter satellite stem (Myf5-negative) cell, whereas the committed daughter (Myf5-positive) cell inherits the Notch ligand Delta1 (Kuang et al., 2007). Accordingly, the Notch antagonist Numb was shown to be asymmetrically located in the committed Myog-positive daughter cell (Conboy and Rando, 2002; Conboy et al., 2005). These results are consistent with the role of Notch in promoting satellite cell return to quiescence and self-renewal (Gopinath et al., 2014; Pisconti et al., 2010; Wen et al., 2012). After asymmetric division, the ability of the two daughter cells to activate the myogenic program is also controlled by Pax7 transcriptional activity. Pax7 was shown to recruit the histone methyltransferase complex Wdr5-Ash2l-Mll2 (Kmt2) to the *Myf5* locus, resulting in permissive chromatin modifications that stimulate transcriptional activation of *Myf5* (McKinnell et al., 2008). However, to recruit this histone methyltransferase complex, Pax7 must be methylated by the arginine methyltransferase Carm1 (Kawabe et al., 2012). During asymmetric division, interaction between Carm1 and Pax7 in the nucleus of the committed daughter cell activates the transcriptional expression of *Myf5* (Kawabe et al., 2012).

Other types of asymmetric divisions and asymmetric segregations of factors have also been described in myoblasts (Fig. 3) (Wang et al., 2013). For example, MyoD was shown to be able to asymmetrically distribute in the two daughter cells, giving rise to one Pax7^+^ MyoD^−^ reserve cell and one Pax7^−^ MyoD^+^ committed myogenic cell (Liu et al., 2012). Similar results were obtained with Myog, with myoblasts generating one Pax7^+^ Myog^−^ and one Pax7^−^ Myog^+^ daughter cell (Yennek et al., 2014). Asymmetric segregation of template DNA has also been proposed as a model to measure asymmetric division in activated satellite cells. Indeed, using transgenic Pax7-nGFP it was shown that, whereas activated satellite cells expressing low levels of Pax7 (Pax7^Lo^) perform random DNA segregation, those expressing higher levels of Pax7 (Pax7^Hi^) mostly perform asymmetric DNA segregation during cell division (Rocheteau et al., 2012). The daughter cell inheriting the old template DNA retains Pax7 expression, whereas that receiving the new template DNA expresses Myog (Yennek et al., 2014). Asymmetric DNA segregation measured by BrdU incorporation showed that it is also associated with the asymmetric inheritance of cell fate determinants, with the daughter cell that inherits the old template DNA expressing SCA-1 (Ly6a) and the cell that receives the new template DNA being positive for desmin (Conboy et al., 2007).

The capacity of satellite cells to choose whether to perform symmetric or asymmetric division allows them to coordinate their activity with the needs of the regenerating muscle. For example, an increased proportion of symmetric divisions would promote expansion of the satellite stem cell pool, whereas increased asymmetric divisions would favour the generation of myogenic progenitors and maintenance of the stem cell pool (Fig. 3) (Wang et al., 2014). Thus, a dynamic balance must exist between symmetric and asymmetric divisions that fluctuates during the different stages of muscle regeneration. Conversely, one could hypothesize that a prolonged imbalance in the symmetric:asymmetric division ratio will lead to impaired muscle regeneration. A better understanding of the mechanisms driving satellite cell fate decisions is needed to determine whether intrinsic defects in satellite cells are involved in pathological conditions.

### Establishing polarity

Asymmetric divisions are controlled by two major interacting events: (1) the unequal distribution of polarity proteins and cell fate determinants; and (2) mitotic spindle orientation. How these processes are regulated in satellite cells remains largely elusive, although recent studies highlight a role for members of the Partitioning-defective protein (PAR) family, which are master regulators of cell polarity establishment in many different stem cell types (Suzuki and Ohno, 2006). In muscle stem cells, recent findings revealed that during asymmetric division the PAR3-PKCλ (Pard3-Prkcι) complex is asymmetrically distributed in the committed daughter cell (Troy et al., 2012). Phospho-p38α/β MAPK also colocalizes asymmetrically in the committed cell with the PAR3-PKCλ complex, where it acts as a determination factor and triggers the activation of MyoD, leading to the commitment of the daughter cell into a myogenic progenitor (Troy et al., 2012). In various stem cell types, polarity establishment has been shown to regulate mitotic spindle orientation and the direction of cell division (Lu and Johnston, 2013). In regenerating muscle, satellite cell symmetric divisions occur mostly in a planar orientation (parallel to the myofiber), whereas asymmetric divisions occur in an apicobasal orientation (perpendicular to the myofiber), suggesting that cell division orientation is a decisive factor in cell fate determination (Kuang et al., 2007).

### The role of the satellite cell niche

The analysis of different adult stem cell populations, such as epithelial stem cells, neural stem cells and haematopoietic stem cells, has revealed that in all these tissues the stem cell niche provides essential cues that influence cell fate decisions (Cheung and Rando, 2013). In skeletal muscle, satellite cells are entrapped between the myofiber and the ECM. In quiescent satellite cells, these two opposing microenvironments lead to the asymmetric distribution of different proteins, including M-cadherin (cadherin 15) and β-catenin that are located on the myofiber side, and ECM-interacting proteins, such as integrin alpha 7, that are located on the basal membrane side. During muscle regeneration, the feedback signals sent by the microenvironment to the activated satellite cells are important to establish cell division orientation, which is a key factor in determining daughter cell fate. Real-time imaging of myofibers showed that, for the first cell division, the proportions of apicobasal versus planar divisions are 35% and 65%, respectively (Siegel et al., 2011).

A number of proteins located in the satellite cell niche have been shown to affect the orientation of division and, hence, cell fate choice. For example, collagen VI present in the ECM reduces myogenic commitment and promotes the self-renewal of activated satellite cells (Urciuolo et al., 2013), and its deletion strongly impairs the maintenance of satellite cell number following multiple muscle injuries. Fibronectin, which is transiently expressed by activated satellite cells during muscle regeneration, also promotes expansion of the satellite stem cell population. Accordingly, knockdown of fibronectin severely reduces satellite cell engraftment and self-renewal potential (Bentzinger et al., 2013). Fibronectin functions by binding to syndecan 4 (Sdc4), which is located on the satellite cell membrane and forms a co-receptor with the frizzled 7 (Fzd7) receptor. The wingless family member Wnt7a, a soluble factor released in the satellite cell niche by regenerating myofibers, also binds to Fzd7-Sdc4-fibronectin and, together, this complex stimulates the symmetric division and expansion of the satellite stem cell subpopulation (Le Grand et al., 2009). Intracellularly, the binding of Wnt7a to Fzd7 activates the small Rho-GTPase Rac1, which is a member of the PCP pathway, leading to the symmetric polarization of different PCP effectors, such as Vangl2, at opposite poles of the cell along the myofiber axis (Le Grand et al., 2009; von Maltzahn et al., 2012). Activation of the PCP pathway leads integrin alpha 7 to colocalize with Vangl2 on both ends of the dividing cell, thereby allowing the two daughter cells to bind to laminin (Le Grand et al., 2009). Together, these various components of the satellite cell niche promote the symmetric planar division of satellite cells, allowing the two daughter cells to receive similar feedbacks from their microenvironment by remaining in contact with both the myofiber and the ECM.

Conversely, asymmetric divisions take place in an apicobasal orientation, such that the satellite stem cell remains attached to the basal membrane while the committed satellite cell is in contact with the myofiber (Kuang et al., 2007). The daughter stem cell and the daughter committed cell are therefore in physical contact with distinct microenvironments that differently influence cell fate decisions. During apicobasal divisions, the satellite stem cell (Myf5 negative) remains attached to the basal lamina, while the committed progenitor (Myf5 positive) is pushed towards the myofiber and retains no contact with the basal lamina. Notably, during muscle regeneration, satellite cells can traverse the basal lamina and locate in interstitial areas (Ogawa et al., 2014). The influence of ECM binding on cell fate decisions was also studied in myoblasts cultured *in vitro* on symmetric or asymmetric micropatterns coated with fibronectin and fibrinogen (Yennek et al., 2014). Myogenic cells submitted to ECM in asymmetric adherence motifs (such that one side of the cell has higher adherence to ECM proteins than the other) are more prone to perform asymmetric division (Yennek et al., 2014). Interestingly, the subpopulation of Pax7^Hi^ satellite cells that intrinsically tends to perform asymmetric divisions is not affected by symmetric or asymmetric micropatterns, indicating that intrinsic factors also predetermine satellite cell fate (Rocheteau et al., 2012; Yennek et al., 2014).

Overall, cell fate decisions in satellite cells involve a finely tuned balance between intrinsic and extrinsic factors. The tight regulation of this balance is essential for optimal muscle regeneration, and any perturbations might lead to diverse muscle pathologies. The satellite cell niche provides crucial feedback to establish cell polarity and orient cell division. Intrinsically, the symmetric or asymmetric distribution of cell fate determinants also influences the status of the daughter cells. However, the exact mechanisms by which polarity is established in satellite cells remain to be characterized.

### Metabolic control of satellite cells

Quiescent satellite cells have a low metabolic rate, but their activation and entry into the cell cycle are characterized by major metabolic changes. Isolated satellite cells cultured *in vitro* experience a switch from oxidative to glycolytic metabolism (Ryall et al., 2015). Interestingly, emerging studies indicate a role for metabolic pathways in satellite cell fate decisions. The switch to glycolytic metabolism observed after satellite cell activation was shown to decrease the levels of NAD^+^, which in turn decreases the activity of the histone deacetylase sirtuin 1 (Sirt1) and increases H4K16 acetylation, ultimately leading to the activation of muscle gene transcription (Ryall et al., 2015). Pax7-specific *Sirt1* knockout mice show premature differentiation of activated satellite cells and impaired muscle regeneration (Ryall et al., 2015). Moreover, Sirt1 also regulates the autophagy process that provides the nutrients to generate ATP and sustain high energy demands during satellite cell activation (Tang and Rando, 2014). Consequently, *Sirt1* knockout or autophagy inhibition results in impaired satellite cell activation (Tang and Rando, 2014). Oxygen availability also regulates satellite cell fate decisions, and myoblasts cultured in hypoxic conditions exhibit increased Notch signalling that, in turn, promotes high levels of Pax7 expression (Liu et al., 2012). Therefore, myoblasts cultured in hypoxic conditions have decreased differentiation ability but enhanced self-renewal potential, leading to higher transplantation efficiency (Liu et al., 2012). Similarly, satellite cell transplantation efficiency is increased following calorie restriction in either the donor or the recipient mice (Cerletti et al., 2012).

Together, these studies indicate that the metabolic status of satellite cells regulates key pathways involved in cell fate decisions. However, the metabolic modifications observed in satellite cells can affect various molecular signalling pathways in the cell. Therefore, additional studies into the molecular events following metabolic changes in satellite cells are needed and will likely lead to the identification of novel cellular targets that regulate satellite cell behaviour.

## Changes in satellite cells during aging

Both skeletal muscle mass and strength gradually decline with aging (Frontera et al., 2000). Reduced myofiber size is observed in combination with the accumulation of intramuscular fat, fibrosis and chronic inflammation, suggesting that aged muscles exhibit impaired regeneration potential (Barani et al., 2003; Grounds, 1998). Previous studies suggested that the decreased numbers of satellite cells observed in aged muscles could partially explain this reduced regenerative potential (Shefer et al., 2006). However, the extent to which the satellite cell population is reduced is still debated and varies considerably between studies and animal models (Brack and Rando, 2007). Moreover, the density of satellite cells remains relatively stable between aged (20-24 months) and very old or ‘geriatric’ (28-32 months) mice, whereas the numbers and size of the myofibers decline sharply during the same period. Therefore, accumulating evidence indicates that it is the intrinsic regenerative potential of satellite cells, together with the satellite cell niche, that is impaired in old muscles.

### Impaired self-renewal of aged satellite cells

The exact nature of the defects observed in aged satellite cells is beginning to emerge. For example, *in vitro* experiments have shown that activation takes longer in aged satellite cells, but that cell cycle progression is not affected (Barani et al., 2003). Moreover, timecourse measurements of the expression of myogenic regulatory factors after cardiotoxin injury indicate that MyoD and Myog levels remain elevated at later time points in aged as compared with young muscles (Marsh et al., 1997). These results suggest that the commitment of satellite cells is unaffected in aged muscles but that self-renewal and return to quiescence could be impaired. Accordingly, the analysis of markers of cell proliferation, such as BrdU incorporation and Ki67 staining, confirmed that aged satellite cells have increased cycling activity in resting conditions (Chakkalakal et al., 2012). Although most satellite cells show impaired self-renewal in aging, a small subpopulation of aged satellite cells seems to maintain its ability to self-renew, suggesting that a stem cell subpopulation could have intrinsic resistance to aging (Collins et al., 2007).

**Fig. 4.**
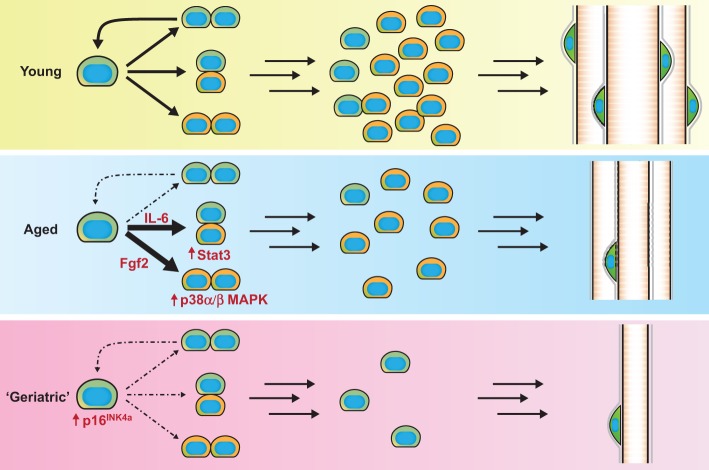
**Satellite cells in aging.** In young muscle, a critical balance between lineage commitment and self-renewal is maintained during regeneration. By contrast, aged muscles show increased lineage commitment (solid arrows) to myogenic progenitors (orange cells) and a lack of self-renewal (dashed arrows), resulting in impaired regeneration and slow exhaustion of the satellite cell reserve (green cells). Satellite cells from very old (‘geriatric’) muscles enter senescence and lose their ability to re-enter the cell cycle.

The mechanisms driving this inability of old satellite cells to maintain or return to quiescence and to self-renew have been extensively studied in recent years. The expression of Fgf2, which is involved in satellite cell activation, was shown to be upregulated in the aged satellite cell microenvironment, whereas the FGF signalling inhibitor Spry1 is downregulated (Chakkalakal et al., 2012). Interestingly, inhibition of FGF signalling rescues the self-renewal capacity in old satellite cells (Chakkalakal et al., 2012). p38α/β MAPK, which acts downstream of the FGF receptor, is also overstimulated in aged satellite cells (Bernet et al., 2014). This overactivation of the p38α/β MAPK pathway induces satellite cell activation and disturbs the asymmetric division of aged satellite cells, leading to the increased generation of committed progenitors and a reduction in self-renewal (Fig. 4). Furthermore, the activation of p38 MAPK in old satellite cells cannot be rescued by transplantation into a young myofiber, indicating that it is a deficit inherent to the aged satellite cells that cannot be overcome by the satellite cell niche (Bernet et al., 2014; Cosgrove et al., 2014). However, pharmacological inhibition of p38α/β MAPK in aged satellite cells is able to restore engraftment potential and improve their self-renewal ability.

Genome-wide expression analyses of freshly isolated satellite cells from young or old muscles revealed that JAK/STAT is another signalling pathway upregulated in aged satellite cells (Price et al., 2014). *In vitro* experiments showed that Jak1-Stat1-Stat3 signalling promotes myoblast proliferation and represses premature differentiation by regulating the cell cycle inhibitors p21^Cip1^ and p27^Kip1^ (Sun et al., 2007). High levels of the inflammatory cytokine IL-6 in aged serum are partially responsible for this increased JAK/STAT signalling (Fig. 4) (Tierney et al., 2014). Consequently, aged muscle satellite stem cells preferentially undergo asymmetric divisions, and this predisposition can be reversed by treatment with siRNA targeting *Jak2* or *Stat3* (Price et al., 2014). Interestingly, this bias and the resulting lack of symmetric divisions could partially explain the inability of aged satellite cells to self-renew. Accordingly, JAK/STAT inhibition in isolated myofiber cultures increases the number of Pax7^+^ satellite cells in aged mice and decreases the number of committed Pax7^−^ MyoD^+^ myogenic progenitors (Price et al., 2014; Tierney et al., 2014). Pharmacological inhibition of the JAK/STAT pathway also strongly improves the engraftment potential of young and aged satellite cells. Moreover, the direct injection of JAK/STAT inhibitors into regenerating muscle improves myofiber size, satellite cell numbers and the recovery of muscle force (Price et al., 2014).

Altogether, these results indicate that the ability of satellite cells to maintain/return to quiescence and self-renew is impaired in aged satellite cells. More importantly, the discovery of dysregulated intracellular pathways involved in these defects opens up new therapeutic avenues. However, pharmacological inhibitors of the p38 MAPK or JAK/STAT pathways that promote satellite cell self-renewal must be used in a transient manner to replenish the satellite cell population without affecting muscle regeneration. For instance, genetic deletion of *Stat3* in satellite cells improves their expansion but impairs myogenic differentiation, whereas the transient downregulation of *Stat3* increases both satellite cell density and muscle fiber size (Tierney et al., 2014). Nonetheless, pharmacological inhibitors provide a proof-of-concept that the regenerative potential of aged satellite cells can be rejuvenated.

### Modifications in the aged satellite cell niche

Whether the defects in the regenerative capacity of aged muscle are caused by changes in the satellite cell niche or whether they are intrinsically inherent to aged satellite cells is still under debate. However, a number of studies have shown that modifications to the satellite cell microenvironment, resulting from local or systemic changes, are at least partially responsible for the impaired satellite cell regenerative potential of old muscles. For example, *in vitro* experiments showed that the addition of young serum to old myoblasts improves their myogenesis potential (Carlson and Conboy, 2007). Likewise, *in vivo* experiments indicated that the exposure of satellite cells to a young environment partially rescues their regenerative potential (Carlson and Faulkner, 1989; Conboy et al., 2005).

The expression of a number of different cytokines and growth factors is modulated in aged muscles. TGFβ (Tgfβ1), for example, is one factor that is overexpressed in old muscle and that induces phosphorylation and activation of Smad3 in aged satellite cells (Carlson et al., 2008). Smad3 activation, in turn, upregulates various cyclin-dependent kinase inhibitors, including p15, p16, p21 and p27. Importantly, a reduction in TGFβ/Smad3 activation restores the regenerative potential of old satellite cells (Carlson et al., 2008). Interestingly, Smad3 activation directly antagonizes the Notch signalling pathway, which is known to regulate satellite cell quiescence and self-renewal (Bjornson et al., 2012; Carlson et al., 2008). Accordingly, decreased expression of the Notch ligand Delta1 and impaired Notch signalling were observed in satellite cells from old regenerating muscles (Conboy et al., 2003). Forced activation of Notch with specific antibodies rescues the regenerative potential of aged muscle (Conboy et al., 2003). Similar results were obtained using heterochronic parabioses (models in which old and young mice share circulatory systems), with young serum restoring Notch signalling and the regenerative potential of old satellite cells (Conboy et al., 2005).

Serum from aged mice also contains a higher concentration of Wnt ligands (Brack et al., 2007). Canonical Wnt signalling directly antagonizes Notch signalling in satellite cells (Brack et al., 2008), and it was shown that the inhibition of Wnt signalling in old muscles restores satellite cell regenerative potential (Brack et al., 2007). Moreover, high expression levels of Wnt in aged serum force the conversion of myogenic cells toward the fibrogenic lineage (Brack et al., 2007). Interestingly, many ECM components physically interact with satellite cells, and the excessive fibrosis observed in aged muscle is thus likely to perturb satellite cell behaviour. Altogether, these results indicate that changes in the aged satellite cell niche are partially responsible for the impaired regenerative potential of aged muscle.

### Satellite cell senescence

Consistent with the inability of aged satellite cells to maintain quiescence it was demonstrated in geriatric mice (30 months old) that a proportion of satellite cells displays a loss of reversible quiescence (pre-senescence) (Sousa-Victor et al., 2014a). Under proliferative pressure induced by muscle injury, satellite cells from geriatric muscles thus fail to activate and proliferate and instead undergo full senescence and irreversibly withdraw from the cell cycle (geroconversion) (Sousa-Victor et al., 2014b). The strong upregulation of the cyclin-dependent kinase inhibitor p16^INK4a^, an alternative spliced variant of p19^Arf^ (Fig. 4), is at least partially responsible for this loss of reversible quiescence (Sousa-Victor et al., 2014a). This geroconversion further reduces the self-renewal and regenerative ability of old muscle satellite cells and cannot be compensated by a youthful environment.

## Concluding remarks and future perspectives

Satellite cells are the protagonists of muscle regeneration. To appropriately fulfil their functions, they must maintain a dynamic balance between their different cell states, namely quiescence, commitment, differentiation and self-renewal. As we have highlighted above, many intrinsic mechanisms are required to regulate the cell cycle and cell fate determination in satellite cells, and dysregulation of these mechanisms results in the loss of regeneration in degenerative conditions, including aging. In particular, aged satellite cells lose their ability to maintain quiescence (through increased cycling or senescence) and, once activated, their cell fate is forced toward commitment to the myogenic lineage in lieu of self-renewal. Whether these changes are caused by modifications in the satellite cell niche or by intrinsic satellite cell defects remains a matter of debate. Some dysfunctions in aged satellite cells can be rescued by a young environment, whereas others cannot, suggesting that both intrinsic and extrinsic deficiencies are present simultaneously.

Many recent studies have shown that, regardless of their origin, these deficits can be overcome by pharmacological inhibitors. These discoveries could represent a cornerstone in the treatment of pathologies such as sarcopenia, an irreversible loss of muscle mass induced by aging that causes serious health issues and currently has no therapy. It is likely that intrinsic perturbations in satellite cell regulatory mechanisms are present in other pathologies, including muscular dystrophies, and further studies are needed to explore these possibilities and to aid our understanding of muscle development, regeneration and degeneration.
